# Depression in the South African workplace

**DOI:** 10.4102/sajpsychiatry.v22i1.814

**Published:** 2016-05-20

**Authors:** Marthinus P. Stander, Margreet Bergh, Helen Elizabeth Miller-Janson, Janetta C. De Beer, Frans A. Korb

**Affiliations:** 1 Hexor (Pty) Ltd, South Africa; 2 Psychiatrist, Private Practice, Johannesburg, South Africa

## Abstract

Depression is a common psychiatric disorder and can be costly, having a significant impact on the individual and employers. The South African Depression and Anxiety Group (SADAG) in partnership with HEXOR, with the support of Lundbeck, undertook research into depression in the workplace, because South African information is not available on this topic. It provides insight into the prevalence of depression within the workplace in South Africa, as well as the impact of depression on the employees and employers in terms of sick leave and levels of productivity, especially when the symptoms include cognitive impairment. It is apparent that stigma plays a pivotal role in the reasons for non-disclosure to employers. It further highlights the magnitude of awareness, early detection and the provision of a holistic support system within the work environment, free from bias, to ensure that optimum benefit can be achieved for both employer and employee.

Findings just released from a research study into the impact of depression on the South African workforce show that at least one in four employees have been diagnosed with depression, that the country’s economic engine age group of 25–44 year-olds is most affected and that during their last depressive episode, employees took 18 days off work due to the condition.

Depression is one of the most common psychiatric disorders and can be costly, having a significant impact on the individual and society. Although clinicians are generally aware of the wide-reaching effects of the symptoms of depression, such as sleep disturbance, anhedonia and suicidal ideation, the influence of this illness on numerous aspects of life is becoming increasingly recognised.

The South African Depression and Anxiety Group (SADAG) in partnership with Hexor, with the support of Lundbeck, undertook research into depression in the work domain because of the many work-related challenges people with depression encounter. During the latter part of 2014, the IDEA survey instrument was administered to 1060 employed or previously employed workers and managers in South Africa. As with the European study, the results provide new details on depression in the workplace that bring to light a situation that warrants attention on many fronts. The initial data analysis indicates that more than 24% (or nearly one in four) of respondents reported that they had previously been diagnosed with depression by a health care professional.

There is growing evidence of the prolonged impact of depression on functioning in the workplace, especially when the symptoms include significant cognitive impairment. It would be in the interest of employers to ensure that affected employees are treated effectively, since their impairment affects the profitability of the organisation. However, there appears to be a surprising lack of awareness of depression and its consequences in the workplace.

The recently completed Impact of Depression in the Workplace in Europe Audit (IDEA) (http://www.europeandepressionday.com/idea.html) explores the impact of depression in the workplace in seven countries in Europe. As highlighted by Evans-Lacko et al.^1^, it begs the question to what extent employers are aware of the significant impact depression has on their workforce, how unequipped managers feel to manage such challenges and how employees respond when they are diagnosed with depression. According to Greenberg et al.^2^, in the United States of America (USA), from 1990–2000, the treatment rate of depression increased by 50%. However, the economic burden (US$ in real terms, i.e. after adjustment for inflation) only increased by 7%. Yet, of the total depression costs in 2000, 62% were attributable to workplace costs (31% were direct medical costs and 7% were suicide-related mortality costs).

Non-disclosure of depression as a reason for sick leave was predominantly due to stigma and work security issues. A significant proportion of respondents also believed that their employer would not know how to support them. Independent of age, level of income, level of education, being a manager or having had a previous diagnosis of depression, most respondents admitted that they feel uncomfortable in dealing with people with depression in the workplace. When confronted with employees with depression or suspected depression, a large proportion of managers were not sure how to respond or responded inappropriately.

In economic terms, the results of this audit highlighted the impact of absenteeism and presenteeism of individuals with depression and, as with the results from the USA, it appears that the most significant cost of depression in the workplace can be attributed to workplace costs.

Other important findings speak to individual or manager characteristics and the likelihood to take off work due to depression. Surprisingly, there’s a uniform behaviour pattern, independent of these characteristics. The same applies to the probability of disclosure that the reason for sick leave was due to depression. Across the board, the disclosure patterns of managers and employees were the same.

When analysing responses only from individuals who reported having previously been diagnosed with depression, important trends emerged. In rating their own work performance with no depression compared to a time when they were suffering from depression, 85% of respondents who gave themselves a top rating in terms of work performance gave a lower rating of their work with depression. Of this subgroup, those who experienced cognitive symptoms had significantly lower job performance scores whilst working with depression compared to those who did not experience cognitive symptoms.

Overall, the South African results paint a picture of fragmented employee support structures and policies related to mental health, specifically depression. The study indicates that the economic burden of depression is high, that stigma impacts negatively on employees’ health-seeking behaviour and that managers do not feel well-equipped to manage employees with depression. The results beg the question whether employers are aware of the burden of depression in their workplace, and of the potential economic and humanistic benefits that can be derived from well-structured employee assistance programmes that address mental health issues. The findings also highlight the important role health care professionals can play in educating patients and possibly employers about depression, the prevalence of which has increased by 50% in the USA over a 10-year period.
